# A Bibliometric Analysis of the Development of ICD-11 in Medical Informatics

**DOI:** 10.1155/2019/1649363

**Published:** 2019-12-25

**Authors:** Donghua Chen, Runtong Zhang, Hongmei Zhao, Jiayi Feng

**Affiliations:** ^1^Department of Information Management, School of Economics and Management, Beijing Jiaotong University, Beijing 100044, China; ^2^Peking University People's Hospital, Beijing 100044, China

## Abstract

The International Classification of Diseases (ICD), which is used to group and report health conditions and factors, provides a basis for healthcare statistics. The 11th revision of the ICD (ICD-11) released by the World Health Organization provides stakeholders with novel perspectives on solving the complexity of critical problems in medical informatics. This study conducts a bibliometric analysis of research published over the period of 1989–2018 to examine the development of ICD-related research and its trends. First, over 4000 ICD-related papers spanning the 30-year period are retrieved from the Web of Science database. Then, based on the meta data of the selected papers, time trend analysis is performed to examine the development of different ICD revisions. Finally, the keywords and topics of these papers are analyzed and visualized using VOSViewer and CiteSpace. Our findings indicate that ICD-11-related research has grown rapidly in recent years compared with studies on ICD-9 and ICD-10. Moreover, the most popular research directions of ICD-11 include the topics psychiatry, psychology, information science, library science, and behavioral science. In terms of perspectives, information system-related research is more common than big data- and knowledge discovery-related work. However, the popularity of big data- and knowledge discovery-related developments has grown in recent years. The use of ICD-11 facilitates the development of medical informatics from the perspectives of information systems, big data, and knowledge discovery.

## 1. Introduction

The International Classification of Diseases (ICD) [1], which was developed by the World Health Organization (WHO), plays a crucial role in governments' reporting, grouping, and statistical analyses of diseases and other health-related factors. The wide use of ICD makes it a global standard for diagnostic health information and enables sustainable and systematic recording, analysis, interpretation, and comparison of mortality and morbidity rates of different countries at different time points. The ICD also covers various signs, symptoms, abnormal findings, complaints, and social factors suitable for studies on financial aspects, such as billing or resource allocation, and provides a basis for big data in personalized healthcare [2]. Moreover, the ICD provides an information framework that allows stakeholders to monitor epidemics and threats toward public health, monitors the expenditure burden shouldered by patients, evaluates the progress in achieving public health objectives, determines the obligation of member states of the WHO to provide free or subsidized medical services to their populations, and develops appropriate healthcare services [3]. Therefore, the ICD is key to the sustainable development of medical big data research [4].

ICD standards have been used in medicine and healthcare for over 100 years. The first ICD standards initially focused on the statistics of the causes of death. In 1946, the Interim Commission of the WHO was entrusted to take over the revision of the ICD and introduced a method for disease classification [5]. At present, the most widely used version of the ICD is its 10th revision (ICD-10), which was endorsed by the Forty-third World Health Assembly in 1990. After over a decade of revisions by numerous countries based on Internet-based maintenance platforms, the WHO released the final version of the 11th revision of ICD (ICD-11) in June 18, 2018, to provide a new de facto standard of disease coding for the twenty-first century [6]. The ICD-11 was submitted to the 144th Executive Board Meeting in January 2019 and then to the 72nd World Health Assembly in May 2019 [7]. Following endorsement, the member states of the WHO are expected to begin reporting on the basis of ICD-11 on January 1, 2022.

The development of new ICD standards is expected to revolutionize global medical informatics within the next few decades. Over the past 20 years, the ICD-10 has been widely utilized to classify healthcare information. For example, ICD-10-coded hospital big data offer new opportunities for monitoring flu epidemics [8]. Numerous ICD-10 national modifications have been developed to adapt actual use in different countries. The ICD-11 at present is ready for testing and implementation in accordance with the specific timelines and requirements of different countries [9]. The structure and design of the newly proposed ICD-11 are based on clinical practices over the past few decades and differ considerably from those of its previous revisions [10]. ICD-11-coded medical records provide the basis of massive health statistics with the latest development of big data-driven intelligent healthcare using big data analytical platforms such as Apache Hadoop and Spark [11, 12]. However, the increasing use of ICD-11 in medical and health big data reduces the applicability and relevance of past analytical methods because the ICD-11 features new code schemes and concepts that differ from previous ICDs, such as stem codes representing entities or groupings of high relevance or clinical conditions that should always be described as a single category. Appropriate utilization of ICD-11 for the analysis of mortality, morbidity, epidemiology, case mixing, quality and safety, primary care, and detailed information from medical and health big data are essential to provide the basis for big data research in health informatics [13].

Introduction of the novel concepts of ICD-11 can overcome the problems of previous ICD revisions. The foundation component and content model are key concepts in ICD-11. The foundation component is a multidimensional collection of all ICD entities. The content model describes several specific diseases or disorders and is defined by 13 attributes, namely, ICD entity title, classification properties, textual definitions, terms, body system/body part, temporal properties, subtype property severity, manifestation properties, causal properties, functioning properties, specific condition properties, treatment properties, and diagnostic criteria. The content model also illustrates background knowledge that provides the basis for the systematic definition of each ICD entity to enable computerization. New disorders, such as gaming disorder, which remains controversial, are introduced in ICD-11 [14]. In contrast to ICD-10, ICD-11 is established on the basis of ontology models [15]. Several value sets in ICD-11 are derived from external ontologies, such as the Systematized Nomenclature of Medicine–Clinical Terms (SNOMED CT) [16], which has played an important role in healthcare. Novel concepts in ICD-11, such as stem codes and post-coordination, are proposed to overcome the challenges encountered by ICD-10 in recent years because the latter is now outdated in the clinical and classification points of view. For example, stem codes containing all pertinent information in a precombined fashion in ICD-11 are referred to as “pre-coordination;” when additional detail pertaining to a single condition is described by combining multiple codes, the code combination is referred to as “post-coordination.” ICD-11 also allows stakeholders to operate in an electronic environment and capture more information, especially for morbidity use cases. In summary, the newly proposed ICD-11 is more suitable for disease coding than past revisions of ICD in the new era of medical informatics.

This study aims to conduct a bibliometric analysis to examine the development of ICD-11-based studies in healthcare. The data sources and search strings are first determined. Time trend analysis is then performed based on the selected papers. Keywords and topics are analyzed and visualized to summarize the main findings of our study from the perspectives of information systems, big data, and knowledge discovery. Finally, we discuss and conclude our work.

## 2. Materials and Methods

**Figure 1:**
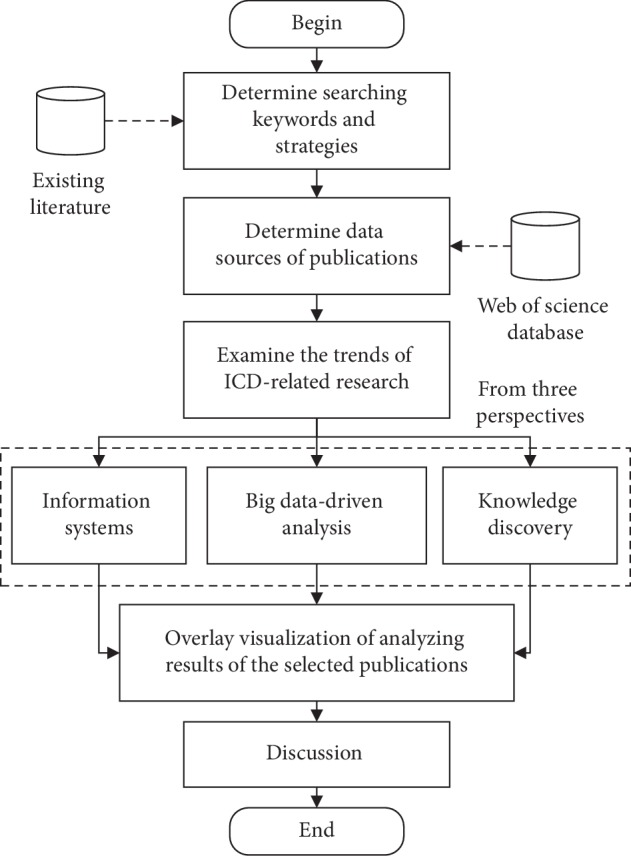
Flowchart of the bibliometric analysis in this study.

Figure 1 illustrates the flowchart of this bibliometric analysis of ICD-related research. First, we determine relevant keywords and conduct search strategies to retrieve ICD-related research. Second, the Web of Science database is used to retrieve relevant publications. Third, time trend analysis of ICD-related papers is performed. Finally, the analysis results are visualized from three perspectives.

### 2.1. Data Sources

The data sources of ICD-related work published over a certain time period are selected to facilitate this bibliometric analysis. Many researchers have applied ICD to their research since 1990, when the ICD-10 was first endorsed. Given that the ICD-9 and ICD-10 have played a crucial role in promoting the development of medical informatics in the last 30 years [17], the trends of relevant studies in this period should be examined. The Web of Science database is used to obtain high-quality papers. However, we acknowledge that the database may not contain several valuable papers in this field. We will synthesize and discuss relevant literature. Relevant articles published over the period of 1989–2018 are retrieved from the Web of Science database by searching the keywords “ICD-9,” “ICD-10,” and “ICD-11” in the article title field in the core set of the Web of Science database. This process can search studies relevant to the ICD national modification because the name of ICD national modifications includes the keyword “ICD.” Table 1 shows the statistics of the publications selected from the database.

**Figure 2:**
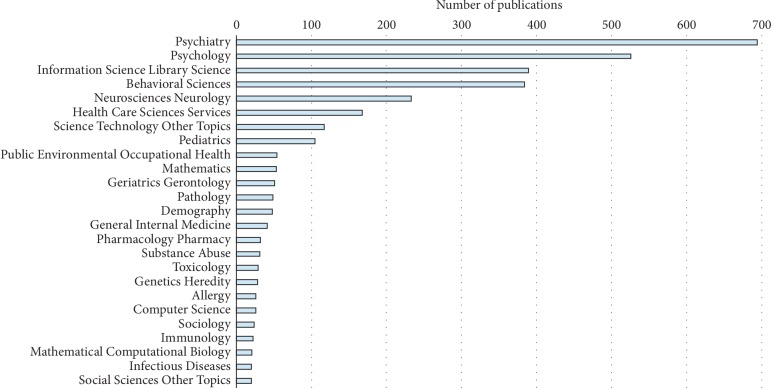
Research directions related to ICD over the period 1989–2018.

The distribution of ICD-related subjects in the selected publications over 30 years is illustrated in Figure 2. Psychiatry and psychology are the most popular subjects in ICD-related clinical research. Information science and library science are other popular research fields that may focus on the development of ICD standards. Behavioral science and neuroscience neurology are related to clinical research. Health science services and science technology are key fields focusing on improvements in the practical use of ICD in medical and health informatics. Research on health science services and science technology is key to intelligent healthcare.

### 2.2. Time Trend Analysis

Time trend analysis aims to examine the development of different revisions of ICD over the past 30 years. First, the trends of publications related to ICD-9, ICD-10, and ICD-11 over the selected period are examined. A timeline view of ICD-related research is then used to analyze ICD-related topics extracted from the keywords of publications retrieved for the period 2009–2018. Finally, three perspectives, namely, medical information systems, big data, and knowledge discovery, illustrate the trends of the number of publications for the past 30 years.

### 2.3. Keyword Analysis

Three perspectives of ICD-related research are examined through overlay visualization of the publications. First, an overlay visualization of 234 publications collected by using the keywords “ICD” and “information systems” from the core set of the Web of Science database is presented to examine studies related to the implementation of ICD in medical information systems. Second, an overlay visualization of 51 publications related to the use of ICD in big data analytics is presented to examine the trends of ICD-related research from the perspective of big data. Finally, an overlay visualization of the existing 54 publications related to ICD and knowledge discovery is presented to investigate the state of knowledge discovery using ICD codes. The distribution of keywords from these three perspectives is examined and discussed.

### 2.4. Topic Analysis

The topics of ICD-related research to promote healthcare are as follows.

#### 2.4.1. Information System Perspective

ICD codes provide the basis of structured medical big data in healthcare. Most work used natural language processing and machine-learning techniques for textual analysis. Without professional clinical inspection, such as evaluation of the proper coding of the clinical statuses of patients, the collected data may be imprecise. ICD codes also enable automated classification of diagnostic terms, such as application of computer-assisted coding in Spanish [18]. The ICD is useful for solving such problems and produces structured data that improve the reliability of results from big data analysis.

#### 2.4.2. Big Data Perspective

ICD codes can be related to different perspectives of big data in healthcare. Analysis of massive individual data from the perspectives of different sources, dimensions, and time points often reveals trends that traditional medical research approaches cannot show [19]. However, the contents of existing medical big data are occasionally incorrect, incomplete, and even unavailable; few datasets are complete and valuable for research purposes. The precision and reliability of analyzing ICD-coded results in big data-driven algorithms rely on the coding quality of ICD when ICD coders encode their medical records [20].

#### 2.4.3. Knowledge Discovery Perspective

The performance of ICD-related analysis generally relies on changes in the main diagnosis in the discharge summary of patients or the accuracy of techniques for extracting information from patient records by medical institutions [21]. Professional and technical requirements for practitioners, especially for fresh coders, are stringent because ICD coders must establish a clear disease classification framework in their mind. Disease-related concepts and relations could be retrieved and associated with other knowledge sources in medical domains with the use of ICD to facilitate clinical knowledge discovery from ICD-coded data [22].

### 2.5. Tools for Visualization

VOSViewer [23] and CiteSpace [24] are used to visualize the search results and examine the key information and trends of publications on the use of ICD.

During network visualization using VOSViewer, items are represented by their label by default by a circle. The size of the label and circle of an item are determined by the weight of the item. The higher the weight of an item, the larger the label and circle of this item. The color of an item is determined by the cluster to which the item belongs. Lines between items represent links. In the overlay visualization using VOSViewer, a color bar is shown at the bottom right corner of the graphic. The color bar is shown only if colors are determined by item scores, which indicates how scores are mapped to colors.

During timeline visualization using CiteSpace, time is mapped to the horizontal position, and clusters are arranged along these horizontal lines. Users can adjust a complex set of parameters to control the analysis process as well as interact and manipulate the visualization of a knowledge domain.

## 3. Results

### 3.1. Trends of ICD-Related Research

**Figure 3:**
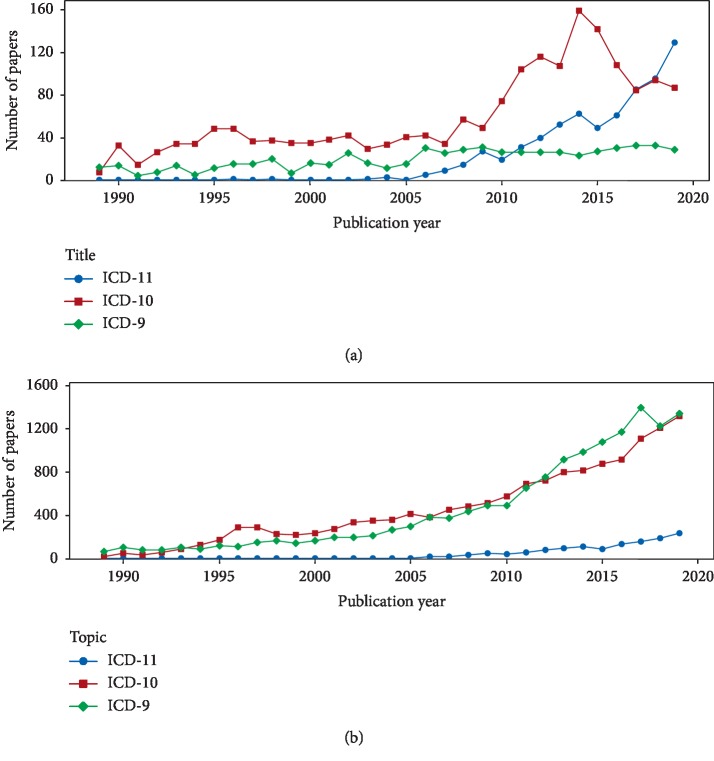
Changes in numbers of publications related to ICD-9, ICD-10, and ICD-11 with publication year according to different search strategies. (a) Publication titles containing ICD-9, ICD-10, and ICD-11. (b) Publication topics containing ICD-9, ICD-10, and ICD-11.

Figure 3 shows the changes in number of publications by publication year using different searching strategies, namely, searching titles of publications containing the keywords “ICD-9,” “ICD-10,” and “ICD-11” and searching for publication topics containing the strings “ICD-9,” “ICD-10,” and “ICD-11.”

Figure 3(a) indicates that the number of ICD-11-related papers in 2017 exceeds that of ICD-10-related papers in an analysis by searching titles of publications. The numbers of publications related to ICD-10 gradually increased over the period of 1989 and 2018, peaked at approximately 160 publications in 2014, and then rapidly decreased to 87 in 2019. However, the number of ICD-9-related research each year remained stable between 20 and 30. The figure shows that ICD-11 has become the focus of ICD-related studies.

Figure 3(b) indicates that the numbers of publications related to ICD-9, ICD-10, and ICD-11 approximately increased over each publication year in an analysis by searching topics of publications. The number of publications related to ICD-11 each year is much smaller than those of ICD-9- and ICD-10-related research. The number of ICD-9-related research publications exceeded that of ICD-10-related research between 2012 and 2018. The number of publications related to ICD-11 continually increased to over 200 in 2019. ICD-11 related research may include keywords of ICD-9 and ICD-10. Overall, research topics on ICD-11 and, in turn, the number of relevant publications, began to show an upward trend in 2006.

**Figure 4:**
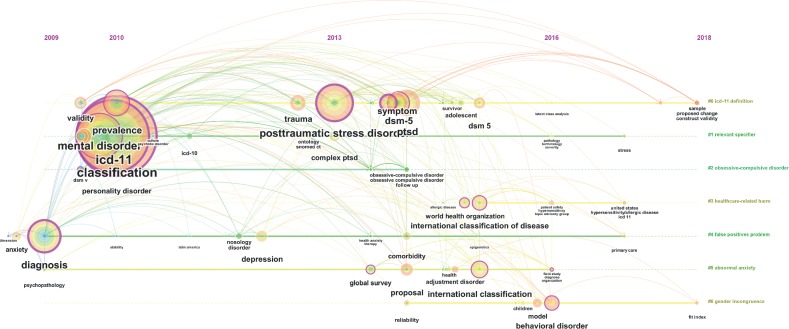
Timeline view of ICD-related research over the period of 2009–2018.

Figure 4 depicts a timeline view of ICD-related research trends over the period of 2009–2018 by using CiteSpace. The visualization results in the figure demonstrate the ICD-related topics extracted from the keywords of the retrieved publications from 2009 to 2018. A larger circle in the figure indicates a higher popularity of the corresponding topics in the year; conversely, a small circle indicates that the keyword-related research is less popular. The topics in the figure are clustered into seven groups, namely, “ICD-11 definition,” “relevant specifier,” “obsessive–compulsive disorder,” “healthcare-related harm,” “false positive problem,” “abnormal anxiety,” and “gender incongruence.”

**Figure 5:**
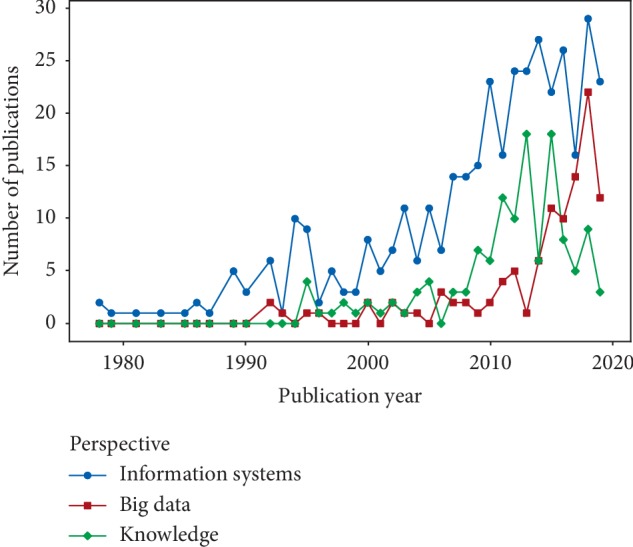
Trends of the numbers of publications related to information systems, big data, and knowledge discovery.

Figure 5 illustrates the trends of the number of publications related to the three perspectives discussed earlier. Results indicate that, over the last 30 years, ICD has been more extensively used in information systems than in big data and knowledge discovery. The number of publications related to information systems, big data, and knowledge discovery increased roughly each year but studies on information systems were published much earlier and with greater frequency than studies on big data research and knowledge discovery. The trends of ICD-related research may be expected to play a crucial role in big data analysis based on ICD-coded data and knowledge-based systems.

### 3.2. Distribution of Keywords

The distribution of keywords in this bibliometric analysis is summarized in Table 2. The Total Link Strength (TLS) attribute in the table indicates the number of links of an item with other items and the total strength of the links of an item with other items, respectively. The tabular results show the top 10 keywords involved in the three perspectives according to our analysis using VOSViewer. Network visualizations of the keywords in different perspectives also follow.

### 3.3. Perspective of Information Systems

**Figure 6:**
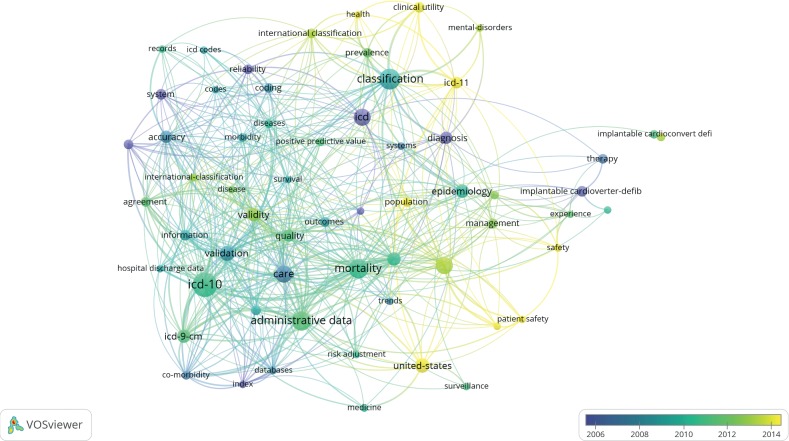
Overlay visualization of 320 publications related to the use of ICD in information systems published over the period of 2006–2018.

The overlay visualization with respect to the perspective of information systems is shown in Figure 6; Table 2 (Part A) shows the statistics of popular keywords in medical information systems. First, the network indicates that studies related to information systems are often associated with research on administrative data (18/234), classification (20/234), and mortality-related data (18/234). Second, ICD-11-related research is becoming a popular trend and often related to patient safety, population, mental disorders, and clinical utilities, although ICD-10 also provides a broad research foundation that covers various needs in medical information systems (26/234). Third, the network visualization illustrates major research directions that future testing and implementation of ICD-11 should follow. Finally, the visualized results show the trends of the transition of use from ICD-9 and ICD-10 to ICD-11. Figure 6 shows that the publication years of ICD-9- and ICD-10-related studies (green lines) are between 2008 and 2012 and that the publication years of ICD-11-related studies (yellow lines) are between 2013 and 2018.

The visualization presented in Figure 6 implies that the adaptation process for ICD-11 will be more efficient than that for ICD-10 despite the 10 years required to develop the clinical modification of ICD-10. The politicking and issues encountered over the past 10 years need not be repeated.

### 3.4. Perspective of Big Data

**Figure 7:**
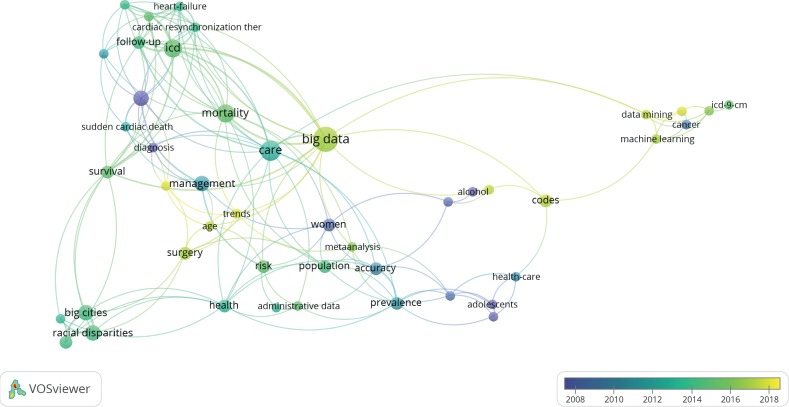
Overlay visualization of 51 publications related to the use of ICD in big data over the period of 2008–2018.

Table 2 (Part B) shows the statistics of popular keywords in big data research. The number of publications related to big data-related research is smaller than that related to the use of information systems, as shown in Figure 7. First, big data-related research (8/51) with ICD is a promising field because the concept of big data in research only emerged after 2016. Second, big data research is associated with data mining, machine-learning algorithms, and management, whereas past research aspects focused on clinical research, such as mortality (5/51), breast cancer (3/54), adolescents, and risk. Third, connections (5/51) exist between big data and ICD; these connections include statistics of mortality (5/51) and healthcare (3/51). Other additional valuable information can be found in Figure 7.

The visualization in Figure 7 provides the past and present trends of research and their connections to the development of different versions of ICDs from different perspectives. The results encourage the use of ICD, especially ICD-11, in big data-driven algorithms. Big data-driven algorithms can adopt machine-learning-based methods that enhance the statistics of ICD-11-coded big data for future mortality and morbidity research.

### 3.5. Perspective of Knowledge Discovery

**Figure 8:**
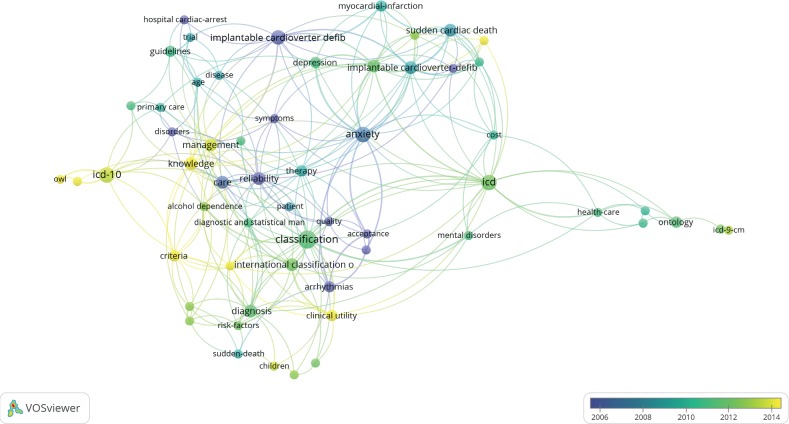
Overlay visualization of 54 publications related to the use of ICD in knowledge discovery.

Table 2 (Part C) shows the statistics of popular keywords related to knowledge discovery. First, the results in Figure 8 show that studies on knowledge discovery are usually related to the analysis of ontology-based models for prediction, hospital management (4/54), and quality of life (4/54). Second, the use of ICD-10 in knowledge discovery is currently a hot spot; thus, ICD-11 may be expected to become increasingly popular in the future. Third, external knowledge sources (4/54), such as guidelines and databases in the medical field, are necessary for knowledge discovery from medical records. Third, most research related to knowledge discovery is disease based. Therefore, ICD-based knowledge discovery in healthcare is insufficient but promising for improving predictions and risk management from the clinical perspective.

The visualization analysis in Figure 8 indicates that the number of publications related to knowledge discovery is smaller than that related to the implementation of information systems and big data-driven analysis. The results provide new perspectives on the use of the newly designed ICD-11 to enhance knowledge discovery in medical big data.

## 4. Discussion

This study conducts a bibliometric analysis of research published over the period of 1989–2018 to examine the development of ICD-related research and its trends. The time trend analysis indicates that ICD-11 related research has grown rapidly in recent years compared with ICD-9 and ICD-10 studies and that the popular research directions of ICD-11 include the topics psychiatry, psychology, information science, library science, and behavioral science. In terms of perspective, information system-related research is more common than big data- and knowledge discovery-related work. Information system research is associated with keywords including “administrative data,” “ICD-10,” “mortality,” and “validation.” However, trends also show that big data- and knowledge discovery-related research has become more popular in recent years. Big data-related research is associated with keywords such as “healthcare” and “mortality,” while knowledge discovery-based research is related to keywords such as “quality-of-life,” “management,” and “anxiety.” The use of ICD-11 has facilitated the development of medical informatics from the perspectives of information systems, big data, and knowledge discovery.

The release of ICD-11 affects the future implementation of other standards in the medical field [25, 26]. For example, ICD is the most important reference for categorizing disease-related groups in disease-based payment in a hospital. In this case, the necessity and accuracy of ICD coding is highly important. The use of artificial intelligent techniques also frees clinical coders from the burden of coding records [27]. Therefore, developing big data-driven intelligent algorithms that automatically learn massive information from medical record pathological sections and image data to provide guidance for diagnosis and disease treatment and establish different disease models have become more crucial in the era of intelligent healthcare than in the past [28–30]. By extracting and structuring ICD-11-coded data and utilizing expert knowledge, such as ICD-11 and SNOMED CT, the algorithm with the use of ICD-11 could hold potential value for solving critical healthcare problems that cannot be solved by traditional ICD-10.

The ICD was initially established to provide mortality and morbidity statistics [31]. The future use of ICD-11 in healthcare expands the utility of ICD-11-coded big data in healthcare. Facilitating statistical analysis by using ICD-11 for decision-making based on big data is a key concern of governments and the WHO because these institutions may require an overview of healthcare data to improve national healthcare policies and provide early warnings for diseases and risks [26, 32]. The information systems of hospitals will likely entail multiple upgrades to support the transition from ICD-9 and ICD-10 to ICD-11. Numerous reasons for these upgrades may be cited. First, ICD-11 adoption may require considerable technological modifications, such as ontology modeling for IT vendors, trading partners, external reporting entities, and third-party payors. Second, productivity loss is anticipated in functional areas that routinely use ICD-9 and ICD-10 codes. Third, training programs for new/revised clinical documentation requirements and coding nomenclature should be developed for coders, medical staff, nurses, and allied health providers (e.g., respiratory, physical, and occupational therapists). The WHO has developed ICD-11 Application Programming Interfaces (ICD-API) and its container version of the ICD-API to support ICD-11 implementation in hospitals [33]. Fourth, physician practices may face financial and operational burdens from ICD-10 implementation and other technological requirements. Finally, the move from the diagnosis and procedural codes of ICD-9 and ICD-10 to those of ICD-11 may raise concerns about protected health information security and privacy risks.

Automated ICD coding for medical records based on diagnostic information is the most popular research direction taken by ICD coding experts to improve the efficiency and accuracy of ICD coding in hospitals. Automated coding is a complicated computer-aided process involving numerous task-oriented algorithms, such as natural language processing techniques [34] and semantic web technology [35], that allow utilization of ICD coding rules to support coding of medical records. The WHO provides us with a simple ICD-11 coding tool online to demonstrate the use of ICD-11 coding [36]. The accuracy of ICD coding tasks mainly relies on the abilities of coders from the medical record department. These coders can code the summary of diagnosis in electronic health records after patient discharge. If the coders have questions related to a patient's records, they will ask doctors to clarify the information to maintain medical record quality. However, inexperienced ICD coders produce medical records with poor coding quality that, in turn, provide poor-quality big data for analysis [37]. Occasionally, several semantic web-based approaches for harvesting multilingual textual definitions in ICD-11 must be used among different countries [38]. The use of the MapReduce model and proper expert knowledge in automated ICD coding provides high-accuracy and efficient statistics for electronic health records [39]. Thus, novel coding algorithms for ICD-11 are necessary to revise and adapt actual scenes in big data-driven healthcare.

Given that ontology modeling can represent knowledge and support knowledge reasoning in specific fields, an ontology-based ICD is naturally suitable for providing a knowledge base for decision-making in healthcare [40, 41]. Researchers can also develop a customized version of WebProtégé [42] to support the collaborative development of ICD-11 content. Other ontology-based algorithms may also be useful to enhance ICD-11-based decision-making [43]. Therefore, an important use of ICD-11 is the implementation of knowledge discovery in healthcare by transforming big data into healthcare knowledge [44]. Big data-driven algorithms for knowledge discovery using ICD-11 are related to numerous application scenes. For example, researchers may search for a specific diagnostic item in the context of disease classification or establish a conceptual knowledge network from the narratives of electronic health records by using the entities and their relationships found in ICD-11. Through involvement with other big data sources related to healthcare, such as massive medical records, ICD-11-based big data analysis algorithms for knowledge discovery can provide considerable insights into the potential value of big data in healthcare [45]. In these cases, the algorithm usually requires external knowledge sources for ICD-11-based applications.

According to the current analysis, the ICD has been proven to be the most important component of healthcare information systems for clinical research, medical monitoring, and public health management on a global scale [46]. ICD-based statistics from the big data perspective [47] includes the causes of death, diseases, injuries, and symptoms, as well as diagnostic and external disease factors. Hospitals in different regions can use ICD codes to share and compare equivalent medical data and promote medical and financial information management [48]. However, the transition from ICD-10 to ICD-11 complicates the further development of support tools for medical information systems [49–51]. Although the ICD is valuable to research on healthcare-related diseases, implementing this system in each member state of the WHO is difficult. An inevitable tension exists between the incorporation of locally relevant material and the essential purpose of ICD-11, which is to reliably convey clinical information across diverse boundaries [52]. For example, over 20 years since ICD-10 was first released, only about 100 countries have reached ICD-10 standards because the number of codes has increased. In addition, doctors' workloads have increased after adoption of the ICD-11 because patients' diseases, diagnoses, and treatment must be recorded as accurately and as precisely as possible. Medical institutions have had to upgrade their healthcare information systems to adapt to the needs of ICD-11 coding. Substantial time and money are required to hire staff in the fields of medical research, information technology, and administration to complete the transition from ICD-10 to ICD-11 [53].

The limitations of this study are as follows. First, the publication data used in this study mainly come from Web of Science, one of the most reputed indexing databases for publications. The ICD is used worldwide for different purposes, and the outcomes are not always documented as scientific published data. Although several other relevant studies are discussed, other work outside the database may not be fully considered in this study. Second, the main contents of the selected publications were not retrieved for analysis; instead, meta data of the publications were considered in keyword and topic analyses. Third, we focused on studies related to the perspective of medical informatics, rather than clinical perspectives; a thorough review of such medical research may be needed to further analyze findings in the main contents. Finally, the three perspectives used as a basis for analysis were selected subjectively; ICD-11 can provide other implicit uses for medical informatics in the future.

## 5. Conclusions

This study conducts a bibliometric analysis on the development of ICD-related research from three perspectives. The analysis results indicate that ICD-11-related studies have rapidly developed in recent years. Further development of ICD-11-based research is revolutionizing medical informatics. The potential value of the general features, concepts, and code structures of ICD-11 to naturally support big data-driven medical informatics is examined, and findings illustrate the potential uses of ICD-11 in statistical analysis, automated ICD-11 coding, and knowledge discovery in big data in healthcare. The results further suggest that stakeholders should be aware of the future use of ICD-11 to overcome the challenges encountered in earlier implementations of ICD-11 in medical informatics. Substantial time and money are required to hire staff in the fields of medical research, information technology, and administration to complete the transition from ICD-10 to ICD-11.

## Figures and Tables

**Table 1:** Main types of publications retrieved for bibliometric analysis.

Publication type	Number of ICD-9-related publications	Number of ICD-10-related publications	Number of ICD-11-related publications
Article	390	1359	350
Other	178	367	101
Abstract	143	249	112
Meeting	67	89	21
Letter	31	40	31
Editorial	16	83	93
Review	8	116	66
Case report	3	8	1
Clinical trial	3	26	4
News	3	30	9
Reference material	3	8	1
Total	845	2375	789

**Table 2:** Distribution of top 10 keywords in our bibliometric analysis from the perspectives of information systems, big data, and knowledge discovery.

Perspective	Keywords	Occurrence	Total link strength
(A) Information systems	Administrative data	18	82
	ICD-10	26	77
	Mortality	18	72
	Validation	12	67
	Care	15	66
	Classification	20	56
	Complications	10	52
	Quality	9	50
	Accuracy	9	47
	ICD-9-CM	10	47

(B) Big data	Big data	8	14
	Care	6	13
	ICD	5	13
	Big cities	4	10
	Mortality	5	10
	Racial disparities	4	10
	Survival	3	10
	Follow-up	3	9
	Health	3	9
	Breast cancer	3	8

(C) Knowledge discovery	Implantable cardioverter-defibrillator	4	12
	Quality of life	4	12
	Care	4	9
	Classification	8	9
	ICD	6	9
	Knowledge	4	8
	Management	4	8
	Anxiety	6	7
	Sudden cardiac death	4	7
	Diagnosis	4	6

## Data Availability

The data used to support the findings of this study are available from the corresponding author upon request.
